# Plant-Sediment Interactions in Salt Marshes – An Optode Imaging Study of O_2_, pH, and CO_*2*_ Gradients in the Rhizosphere

**DOI:** 10.3389/fpls.2018.00541

**Published:** 2018-05-03

**Authors:** Ketil Koop-Jakobsen, Peter Mueller, Robert J. Meier, Gregor Liebsch, Kai Jensen

**Affiliations:** ^1^MARUM – Center for Marine Environmental Sciences, University of Bremen, Bremen, Germany; ^2^Applied Plant Ecology, Biocenter Klein Flottbek, University of Hamburg, Hamburg, Germany; ^3^PreSens, Precision Sensing GmbH, Regensburg, Germany

**Keywords:** salt marsh, *Spartina*, planar optode, sediment oxygenation, roots, plant–soil interactions, soil chemistry, imaging methods

## Abstract

In many wetland plants, belowground transport of O_2_ via aerenchyma tissue and subsequent O_2_ loss across root surfaces generates small oxic root zones at depth in the rhizosphere with important consequences for carbon and nutrient cycling. This study demonstrates how roots of the intertidal salt-marsh plant *Spartina anglica* affect not only O_2_, but also pH and CO_2_ dynamics, resulting in distinct gradients of O_2_, pH, and CO_2_ in the rhizosphere. A novel planar optode system (VisiSens TD^®^, PreSens GmbH) was used for taking high-resolution 2D-images of the O_2_, pH, and CO_2_ distribution around roots during alternating light–dark cycles. Belowground sediment oxygenation was detected in the immediate vicinity of the roots, resulting in oxic root zones with a 1.7 mm radius from the root surface. CO_2_ accumulated around the roots, reaching a concentration up to threefold higher than the background concentration, and generally affected a larger area within a radius of 12.6 mm from the root surface. This contributed to a lowering of pH by 0.6 units around the roots. The O_2_, pH, and CO_2_ distribution was recorded on the same individual roots over diurnal light cycles in order to investigate the interlinkage between sediment oxygenation and CO_2_ and pH patterns. In the rhizosphere, oxic root zones showed higher oxygen concentrations during illumination of the aboveground biomass. In darkness, intraspecific differences were observed, where some plants maintained oxic root zones in darkness, while others did not. However, the temporal variation in sediment oxygenation was not reflected in the temporal variations of pH and CO_2_ around the roots, which were unaffected by changing light conditions at all times. This demonstrates that plant-mediated sediment oxygenation fueling microbial decomposition and chemical oxidation has limited impact on the dynamics of pH and CO_2_ in *S. anglica* rhizospheres, which may in turn be controlled by other processes such as root respiration and root exudation.

## Introduction

Understanding the dynamics of O_2_, pH, and CO_2_ in rhizospheres is crucial for understanding important ecosystem functions, as these parameters are key drivers of biogeochemical processes involved in carbon and nutrient cycling (Hinsinger et al., 2009). In vegetated waterlogged sediments, steep gradients of O_2_, pH, and CO_2_ build up around roots, demonstrating a particularly pronounced plant control over these parameters (Blossfeld et al., 2013; Koop-Jakobsen et al., 2017; Lenzewski et al., 2018). Plant-mediated sediment oxygenation and rhizosphere acidification are key mechanisms by which wetland plants improve nutrient availability and uptake (Bradley and Morris, 1990; Lai et al., 2012) and weaken the impact of reduced phytotoxins, such as H_2_S, Fe(II), and Mn(II) (Rozema et al., 1985; Lee, 1999; Pezeshki, 2001). In this way, the interaction between roots and their abiotic environment affects key eco-physiological processes controlling primary production in wetlands (Mendelssohn and Morris, 2002).

The abiotic environment of the rhizosphere is controlled through a system of feedback loops between roots, microbes, and sediment chemistry, in which the dynamics of O_2_, pH, and CO_2_ play an essential role (Ehrenfeld et al., 2005). In wetland plants, belowground transport and leakage of oxygen to the sediment increases oxidative acidification lowering pH in the rhizosphere (Begg et al., 1994; Hinsinger et al., 2003) and increases root and microbial respiration, which results in increased CO_2_ production (Morris and Dacey, 1984). Since CO_2_ is a weak acid, this also contributes to a lower pH of the rhizosphere (Hinsinger et al., 2003). Other plant effects are independent of plant-mediated oxygenation. The release of labile organic compounds from roots (i.e., root exudation) exerts a direct control on rhizosphere pH, due to the acidic nature of some of the exuded compounds (Hinsinger et al., 2003). Furthermore, root exudation exerts strong control over microbial activity in the rhizosphere, fueling both aerobic and anaerobic metabolism and consequently CO_2_ production (Megonigal et al., 2004; Wolf et al., 2007; Mueller et al., 2016). Additionally, pH dynamics are affected by nutrient assimilation, as ammonium uptake by plant roots through proton release is the predominant nitrogen acquisition pathway in many wetland plants (Bloom et al., 2002). Ammonium uptake on the other hand is highly affected by sediment oxygenation (Bradley and Morris, 1991).

Plant effects exert strong control over O_2_, pH, and CO_2_ dynamics and their numerous interactions in wetland rhizospheres, with potentially important consequences for carbon and nutrient cycling. However, due to the challenges associated with conducting measurements inside rhizospheres without disturbing the chemical gradients in the process, little is known about the spatial scale and temporal dynamics of O_2_, pH, and CO_2_ and their inter-correlation in wetland rhizospheres. Planar optodes are currently the state-of-the-art technology for spatial and temporal assessment of rhizosphere processes, facilitating imaging of O_2_, pH, and CO_2_ distribution around roots over time (Blossfeld, 2013). Oxygen imaging of plant-mediated sediment oxygenation by planar optodes has slowly gained a foothold in rhizosphere research, in particular in regards to investigations of O_2_ dynamics in waterlogged rhizospheres including wetland vegetation (Askaer et al., 2010; Minett et al., 2013; Koop-Jakobsen and Wenzhöfer, 2015; Larsen et al., 2015; Koop-Jakobsen et al., 2017). In a recent planar optode study of the salt-marsh grass *Spartina anglica*, Koop-Jakobsen and Wenzhöfer (2015) demonstrated the presence of oxic root zones in the rhizosphere, which were spatially restricted to an area around the root tips. Transport of atmospheric oxygen to roots and rhizomes in *S. anglica* allow oxic root zones to persist in darkness, and photosynthetic oxygen production during the day only has a minor contribution to the build-up of oxic root zones (Koop-Jakobsen and Wenzhöfer, 2015). In comparison to planar optode studies of rhizosphere O_2_ dynamics, planar optode-based investigations of pH and CO_2_ are still sparse (Santner et al., 2015). Planar optodes for pH were applied only in a few studies on wetland plants. In waterlogged sediments, planar optode studies of *Juncus effusus* demonstrated significantly lower pH, by up to 0.5 units, along the root tips, due to oxidative acidification (Blossfeld and Gansert, 2007; Blossfeld et al., 2011). In the flood resistant plants *Arundinella anomala* and *Alternanthera philoxeroides*, planar optode studies revealed a pH decrease of up to 0.8 units around growing root tips, which corresponded with excretion of small organic acids (Schreiber et al., 2012). Planar optode studies on CO_2_ dynamics in rhizospheres have not yet been applied on wetland plants, and only two studies have used planar optodes for rhizosphere CO_2_ investigations. Blossfeld et al. (2013) and Lenzewski et al. (2018) demonstrated increased CO_2_ concentrations around individual roots originating from both root and microbial respiration in the legume *Viminaria juncea* and in the freshwater plant *Lobelia dortmanna*, respectively, showing that growing roots exhibit a large zone of influence on sediment CO_2_ content more than 1 cm away from the root surface.

To evaluate how common marsh grasses control the abiotic environment in their rhizospheres, this study investigates the spatiotemporal dynamics of O_2_, pH, and CO_2_ in rhizospheres of *S. anglica*. *S. anglica* is a pioneer species found in the low intertidal zone of many marine wetlands around the world and is known for its ability to modify the abiotic environment of its rhizosphere (Lee, 1999; Koretsky et al., 2008). Using a novel planar optode system (VisiSens TD^®^ from PreSens GmbH), the spatial variation of O_2_, pH, and CO_2_ around roots of *S. anglica* was recorded as 2D images over time. This gave insight into the net effect of multiple processes, such as root respiration, release of low molecular organic acids, plant-mediated oxygen release, microbial respiration, and reoxidation of reduced compounds, all affecting the O_2_, pH, and CO_2_ dynamics inside the *S. anglica* rhizospheres. Investigations of O_2_, pH, and CO_2_ dynamics on the same individual roots during alternating light–dark cycles gave insight into the daily variation and allows for differentiation of the effect of processes influenced by light exposure of the aboveground biomass.

## Materials and Methods

### Sampling and Culturing

Vegetation and sediment samples of *S. anglica* were collected in April in the Danish Wadden Sea at the location “Rømø-dæmningen” (55° 08′44 N, 08°36′57 E), where *S. anglica* inhabits the intertidal zone characterized by silty sediment (mean grain size 13.3 ± 5.0 μm analyzed using a laser diffraction particle size analyzer – Beckman Coulter Company – LS 13320). Square blocks of the *S. anglica* vegetation (20 cm × 20 cm × 20 cm) were dug out and brought to the laboratory, where they were gently divided into smaller pieces. The sediment was washed away from the roots and rhizomes, and samples consisting of 1–2 shoots were separated. The samples were replanted into their native substrate directly in rhizoboxes [aquaria or containers for visualization of rhizosphere processes (Neumann et al., 2009)], prepared for the optode studies. In this study, the rhizobox was constructed from two stacked 1.5-L, deep, square-form plastic pots, where one side of the inner pot was removed (exposable side). This allowed for visual inspection of the belowground biomass by gently separating the pots, while the biomass remained protected from light when the pots were stacked. The rhizoboxes were positioned at an angle of ∼30° in a large tray filled with 20 ppt artificial seawater, which is within the natural range for the Wadden Sea marshes. Angling of the rhizoboxes caused roots to grow along the exposable side of the rhizobox. Samples were cultured in a greenhouse at 20°C with light enhancement for 2 month prior to the optode measurements. 15 rhizoboxes were prepared with samples. However, only in three of these rhizoboxes did root tips grow along the exposable side in a position suitable for optode measurements. This determined our sample size (*n* = 3).

### Preparation for Optode Measurements

Optode measurements on the *S. anglica* samples were performed directly on the rhizobox in which they were grown. Samples with visible growth of roots along the exposable side of the rhizobox were selected for optode investigations. The roots investigated were comparable in size, with an average root diameter (*n* = 3) of 1.13 ± 0.15 mm, measured by analysis of images of the exposed roots. The stacked pots were separated exposing the selected root. An optode foil (4 cm × 4 cm) was placed on the sediment directly over the root, and a transparent plastic cover (4.5 cm × 4.5 cm) was placed on the foil holding it in place and preventing sediment from gliding onto the optode foil. For improved stability, the remaining area of the exposed side (i.e., excluding the optode foil) was wrapped in Parafilm^®^. The optode investigations were conducted with optode foils for O_2_ (PreSens GmbH product code: SF-RPSu4; size 4 cm × 4 cm 0–100% atmospheric O_2_), pH (PreSens GmbH product code: SF-HP5R; size 4 cm × 4 cm range pH: 5–9), and CO_2_ from (PreSens GmbH product code: SF-CD1R; 4 cm × 4 cm, range: 1–25% pCO_2_).

### Optode Measurements

**FIGURE 1:**
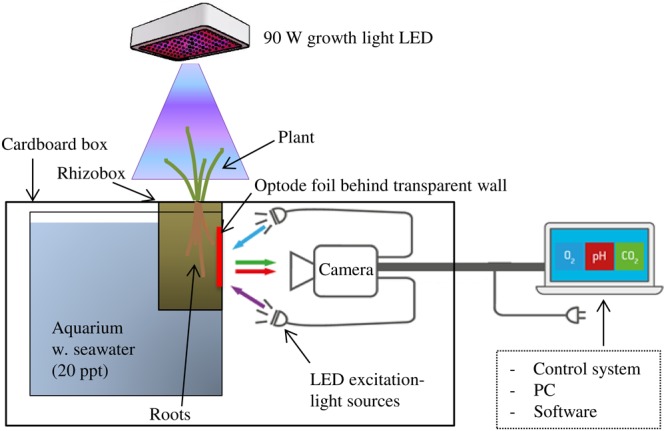
Planar optode set-up for investigation of rhizosphere O_2_, pH, and CO_2_ in waterlogged wetland plants with illumination of the aboveground biomass. The rhizobox was equipped with an optode foil over the root selected for investigation and placed hanging from the top of an up-side-down cardboard box into a water-filled aquarium. Camera for image acquisition and LED lights for excitation of the optode foil were placed in darkness inside the cardboard box.

The principle of optode investigations of O_2_, pH, or CO_2_ is based on either dynamic quenching of a fluorophore in the presence of O_2_ or protonation/deprotonation of a fluorescent dye by the target analyte. Optode foils are coated with an indicator dye containing a fluorophore, which is dynamically quenched by the analyte, and a reference dye containing a fluorophore emitting a stable red or green fluorescent signal. For 2D imaging of rhizosphere processes, an optode foil is placed in direct contact with the roots and sediment, the fluorophores in the foil are excited by a LED light source, and the fluorescent response is recorded with a camera (**Figure 1**). The sensing signal for the target analyte is expressed as a ratio of indicator and reference signal and converted to quantitative measures of O_2_, pH, and CO_2_ through calibration.

In this study, O_2_, pH, and CO_2_ dynamics in *S. anglica* rhizospheres were investigated using the planar optode system VisiSens TD (a novel optode system for mapping of O_2_, pH, and CO_2_ from PreSens – Precision Sensing GmbH, Regensburg, Germany). The VisiSens TD system is a fluorescence imaging-based optode system with integrated hardware for image acquisition and software [VisiSens ScientifiCal (V1.0.0RC38) for image processing that allows read out of O_2_, pH, and CO_2_ sensor foils, respectively]. For a detailed description of the planar optode imaging principles and the theoretical background behind the VisiSens optode system, we refer to Wang et al. (2010) and Tschiersch et al. (2011).

Image acquisition was conducted with a camera resolution of 1292 × 964 pixels facilitating readout of the foils with a high spatial resolution; in this study approximately 14 pixels per mm. Camera and light sources were covered with a large cardboard box (100 cm × 70 cm × 40 cm). The cardboard box was fitted with a hole in the top allowing the rhizobox to hang freely from the top of the box into a water-filled (20 ppt artificial seawater) aquarium placed inside the box, positioning it at a distance of approximately 20 cm from the camera and LED light source (**Figure 1**). In this way, the pathway between the optode foil and the camera was in constant darkness shielded from outside light sources potentially interfering with the fluorescent signal from the optode foil. This set-up facilitated continuous optode imaging of the belowground processes during alternating light exposure of the aboveground biomass.

Measurements were initiated immediately after preparation of the optode foils on the rhizoboxes. However, the initial 3 h period was designated for re-equilibration of the sediment conditions and not included in the final results. During this time, the bulk sediment returned to fully anoxic conditions, and the oxic root zone and CO_2_ and pH changes became visible. All optode measurements were conducted in a temperature controlled climate room at 20°C. In addition to using the integrated VisiSens ScientifiCal-software for image processing, further quantitative image analysis of specific features, such as profiles across roots, were conducted using the image processing software Fiji (Image J, GNU).

Calibration of the optode foils was conducted as prescribed for the VisiSens optode system using the integrated VisiSens ScientifiCal-software. The calibration was conducted in the climate chamber at 20°C; the same temperature at which the experiments were carried out. The O_2_ foil was calibrated using anoxic sediment for 0% O_2_ and air-bubbled seawater (20 ppt) for 100% atmospheric equilibrium [100% atmospheric O_2_]. pH was calibrated using a series of pH buffers in the range from 5 to 8. CO_2_ was calibrated using a series of different gas mixes within the CO_2_ range from 0 to 20vol%. The calibration-gas mixes were produced by mixing known volumes of ambient air with pure CO_2_. The gas mixes were led through a water reservoir prior to entering the calibration chamber resulting in a high degree of water saturation in the air stream in accordance with the calibration guidelines for the CO_2_ foils.

The CO_2_ foils are selective only to free CO_2_ and thereby measure CO_2_ directly as partial pressure in the interstitial water, which in this study was converted to molar concentrations. The other components of the carbonic acid equilibrium, carbonate and bicarbonate, are not included in this measurement. As this study focus on the plant-sediment interaction, it is beyond the scope to account for the entire inorganic carbon budget. To include carbonate and bicarbonate, simultaneous measurements of pH and temperature are required. In general, at the given pH range (6.2–6.6), an experimental temperature of 20°C and a salinity of 20 ppt, CO_2_ accounts for approximately 20–40% of the total inorganic carbon [pK_1_ = 5.96 (Millero et al., 2006)].

### Experimental Design and Data Analysis

In order to capture the spatial and temporal variation of O_2_, pH, and CO_2_ around the roots of *S. anglica*, three roots on three individual plants with root tips growing in a position favorable for optode investigations were selected for the study. For each plant sample, individual optode measurements were conducted for each parameter individually over 2 days with alternating light–dark periods (12/12 h). During light periods, the aboveground biomass was illuminated with a 90 W LED growth light placed at a distance of ∼40 cm above the plant with a radiation of ∼1000 μmol m^-2^ s^-1^ photosynthetically active radiation (PAR), which is above light saturation (Winkel et al., 2011). Optode images were recorded with a temporal resolution of one image per 5 min.

**FIGURE 2:**
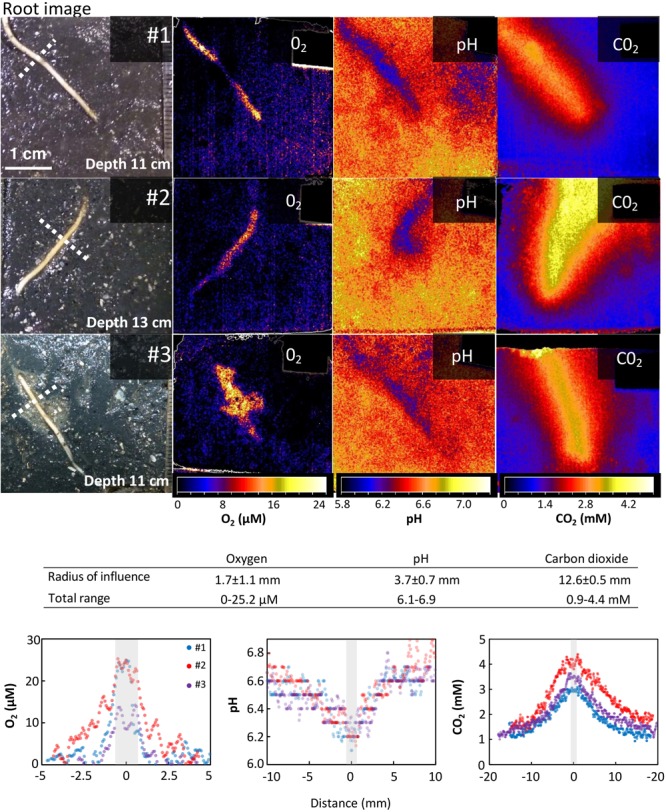
Spatial variation of O_2_, pH, and CO_2_ around roots of *Spartina anglica*. Oxygen release, CO_2_ enhancement, and pH decline are clearly visible in the vicinity of the root. **(Top panels)** Optode images of the spatial distribution of O_2_, pH, and CO_2_ in a 4 cm × 4 cm area around single roots of *S. anglica* from three individual plants (#1-3). Images of the roots and their relative position are shown on the left side (position of root may change slightly between optode images due to root growth and movement during exchange of optode foils). The optode images show the largest impact of roots on O_2_, pH, and CO_2_ in the surrounding sediment observed in these experiments. **(Table inlet)** Table showing the average radius of the area of influence inflicted by roots on O_2_, pH, and CO_2_ in the rhizosphere, and the maximum range of O_2_ and CO_2_ concentrations and pH measured in the profiles. **(Bottom panels)** Cross-sectional concentration profiles of O_2_, pH, and CO_2_. Gray bars indicate relative root position and average root width. Location of profiles are shown by a punctuated line in the root images.

The optode images demonstrate the spatial extent of the impact that the roots exert on O_2_, pH, and CO_2_ in surrounding sediment. Profiles of O_2_ and CO_2_ concentrations and pH were measured perpendicular across the roots (**Figure 2**). The spatial extent of root impact was measured as the distance from the root surface to the point where an effect could no longer be detected. This distance is reported as the radius of influence. Optode images showing the largest effect were selected for this analysis and transversal profiles were measured at the widest position. For O_2_ measurements, spatial analysis was conducted on optode images from the second light period, when the largest effect of light exposure was shown. For pH measurements, spatial analysis was conducted on optode images from the last 15 h after the background pH had stabilized. For CO_2_ measurements, spatial analysis was conducted on optode images selected within the time frame from 8 to 15 h, where the CO_2_ concentrations around the root were largest regardless of the present light conditions.

**FIGURE 3:**
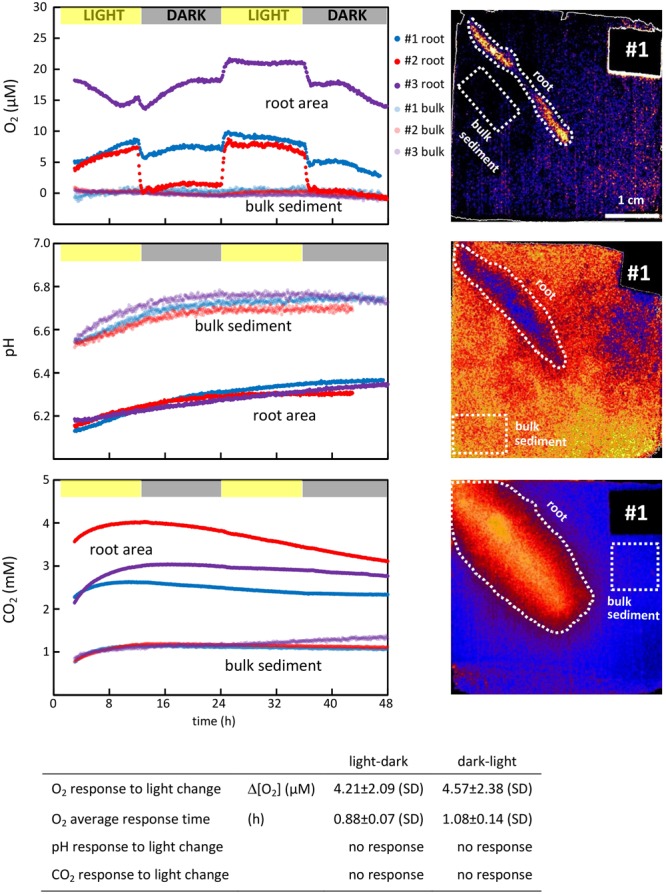
**(Top panels)** Temporal variation of O_2_, pH, and CO_2_ concentrations in the immediate vicinity of *S. anglica* roots and in the bulk sediment. O_2_, pH, and CO_2_ were monitored around single roots from three individual plants during two consecutive light (12 h)-dark (12 h) periods (left). Average O_2_ and CO_2_ concentrations and pH were measured over time within the affected root zone and in a designated area of the bulk sediment. The root zone was selected as the largest area affected by the root, and the bulk sediment was selected in an area unaffected by root presence. Both areas are exemplarily depicted for plant #1 in optode images (right). **(Table inlet)** Table showing the average difference in the rhizosphere concentrations between light and darkness, and the time it takes the rhizosphere concentration to stabilize at a new level.

The measurements over two consecutive light–dark cycles demonstrate the temporal variation in the impact that roots exert on O_2_, pH, and CO_2_ in the surrounding sediment and shows the effect of light exposure of the aboveground biomass. To quantify the temporal variation, the average O_2_ concentrations, pH and CO_2_ concentrations were measured continuously in a designated area around the root and in a designated area in the bulk sediment, where the sediment was unaffected by the root presence (the area selection is exemplified in the optode images in **Figure 3**). The latter represents the background level for O_2_, pH, and CO_2_ in the sediment. This way of measuring temporal variation was conducted using the VisiSens ScientifiCal image processing software, which allows for the average concentration to be followed over time within a manually selected area of the optode foil.

The impact that changing light conditions have on the parameters assessed was measured as the average concentration difference in the designated area around the roots before and after light changes, and is referred to as the average response. The time required for a given parameter to reestablish at a new level after light changes was calculated and referred to as the average response time. These calculations were conducted on selected optode images taken immediately before a light change and immediately after the parameter measured had re-established at a new level (after ∼1 h).

## Results

### Oxygen (O_2_)

For all three plants investigated, O_2_ leakage was present at the root tips (**Figure 2**). In general, the oxic root zones were restricted to a very narrow area in the immediate vicinity of the roots. On average, the oxic root zones had a radius of 1.66 ± 1.14 (SD) mm measured from the root surface to the anoxic bulk sediment, when measured at the widest part. In plant #3, the oxygenated zone comprised a larger area around the lower part of the root tip. This was caused by a textural unevenness in the sediment, which is also visible in the root image (**Figure 2**). Although naturally occurring, it makes the lower part of this root less comparable to the other samples. Hence, the profile was measured at the upper part of the displayed root, where the sediment conditions were undisturbed as in the other replicates (compare root images in **Figure 2**). The maximum O_2_ concentrations measured on the root surface was 21.4 ± 6.3 μM.

All oxic root zones were affected by alternating light–dark conditions (**Figure 3**). The average O_2_ concentration of the oxic root zones immediately decreased when the light exposure of the aboveground biomass was turned off (at 12 and 36 h, **Figure 3**), and immediately increased when the light was turned back on (at 24 h). On average, it took 0.88 ± 0.07 h for the O_2_ conditions around the roots to stabilize at a new level after changes in light condition from light to dark (at 12 and 36 h) and 1.08 ± 0.14 h from dark to light (at 24 h). The magnitude of this response was an average decrease of 4.21 ± 2.09 μM O_2_ from light to dark (at 12 and 36 h) and an average increase of 4.57 ± 2.38 μM O_2_ from dark to light (at 24 h). However, the overall impact of these responses to changing light conditions differed strongly between roots. For plant #1 and #3, the oxic root zone diminished during darkness, but oxic conditions remained in the vicinity of the root. For plant #2, the oxic root zone was eliminated in darkness, resulting in completely anoxic conditions in the sediment surrounding the root. The effect of changing light conditions was characterized by a rapid change in the O_2_ level of the oxic root zones, which was established within approximately 1 h. However, the O_2_ concentrations in the oxic root zones were not stable between light changes, and attenuated fluctuations were observed over time. This was particularly pronounced in plant #3 (**Figure 3**).

### pH

For all three plants investigated, pH was lowered around the roots (**Figure 2**). On average, the radius of the pH-lowered zone was 3.65 ± 0.69 mm, measured from the root surface into the bulk sediment where pH reaches a stable level. A pH minimum of 6.14 ± 0.05 was measured directly on the root surface, which was 0.6 units lower than the average pH of bulk sediment (**Figure 2**). pH-lowered zones were unaffected by alternating light–dark conditions (**Figure 3**), as no pH changes were observed in response to changes in light exposure of the aboveground biomass at 12, 24, and 36 h. During the first 24 h, the sediment was in a re-equilibration phase, counterbalancing the effects on sediment conditions inflicted during installation of the optode foil. This resulted in a steady pH increase of ∼0.2 in the bulk sediment as well as in the root-affected zone. After 24 h, the pH in the bulk sediment stabilized at an average pH of 6.73 ± 0.03 and remained stable at this level during the following light (24–36 h) and dark period (36–48 h). In the root-affected zone, pH continued to increase steadily, reaching an average of 6.34 ± 0.03 at the end of the measurements.

### Carbon Dioxide (CO_2_)

For all three plants investigated, CO_2_ was significantly increased around the roots (**Figure 2**). The affected zone was markedly wider than the affected areas for pH and O_2_, reaching an average radius of 12.6 ± 0.5 mm measured from the root surface into the bulk sediment. A CO_2_ maximum of 3.72 ± 0.63 mM was measured directly on the root surface, which was more than three times higher than the CO_2_ concentration in the bulk sediment (**Figure 2**). The CO_2_-enhanced zones were unaffected by alternating light–dark conditions (**Figure 3**) as no CO_2_ changes were observed in response to changes in light exposure of the aboveground biomass at 12, 24, and 36 h. The CO_2_ concentration in the bulk sediment reached a stable level after 8 h with an average concentration of 1.15 ± 0.05 mM. In the root-affected zone, the CO_2_ level reached a stable maximum level after 8–15 h and subsequently declined slightly.

## Discussion

Overall, this study demonstrated distinct spatial heterogeneity in the distribution of O_2_, pH, and CO_2_ in the rhizosphere of *S. anglica*, and a tight coupling between root processes and O_2_, pH, and CO_2_ gradients was clearly illustrated in the optode images. Changing light conditions had a marked impact on belowground sediment oxygenation, whereas pH and CO_2_ were unaffected by light alterations.

### Spatial and Temporal Dynamics of O_2_, pH, and CO_2_

Oxic roots zones with an average radius of 1.7 mm were clearly visible around the roots, demonstrating that *S. anglica* exerts control of O_2_ in its rhizosphere. The radius of the oxic root zones measured in this study is similar to the radii previously measured in *S. anglica* growing in permeable sandy sediment (average radius 1.4 mm) and markedly larger than in *S. anglica* inhabiting highly reducing tidal flat deposit (average radius 0.35 mm) (Koop-Jakobsen et al., 2017). Differences in the spatial distribution of O_2_ were observed among the studied roots. In plant #3, the oxic root zone was not evenly distributed along the root, due to heterogeneity in sediment texture (**Figure 2**), which was clearly visible in the root image. Such heterogeneity may be facilitated by burrowing infauna or decayed root material.

The oxic root zones of all plants investigated were affected by light exposure, showing an immediate increase in O_2_ concentration in response to light and an immediate decrease in response to darkness. This demonstrates that light regulates belowground sediment oxygenation, likely due to increased photosynthetic O_2_ production and enhanced convective flow. However, the impact of light on the sediment oxygen conditions varied markedly among the studied plants. In two cases (plant #1 and #3), a change from light to dark resulted in a decrease of the O_2_ concentration in the oxic root zone, whereas in one case (plant #2), the oxic root zone was completely eliminated in darkness (**Figure 3**). These observations demonstrate intraspecific differences in the dynamics of belowground sediment oxygenation in *S. anglica*. In a previous study, Koop-Jakobsen and Wenzhöfer (2015) investigated sediment oxygenation in three roots from the same *S. anglica* plant, which all showed the same minor reduction of the oxic root zone when illumination was turned off. This suggests that the oxic root zones are controlled by the O_2_ budget of the whole plant rather than differences among individual roots. This is further supported by the CO_2_ dynamics of the current study, where the plant showing elimination of the oxic root zone in darkness also showed the highest CO_2_ release, indicating higher metabolic activity and thereby O_2_ consumption. The limited number of replicates in this study does not facilitate a full correlation analysis between CO_2_ production and O_2_ release. However, this observation is supported by previous studies showing that root system respiration was the controlling factor in the plant O_2_ balance and release of O_2_ through the roots (Howes and Teal, 1994).

The O_2_ concentration of the oxic root zones did not only vary in response to changing light conditions, attenuated fluctuations were also observed over time irrespective of the light regime. These changes are expected to originate from changes in the gas permeability of root tissue as the root ages, which was previously shown in other wetland plants (Armstrong, 1971; Frederiksen and Glud, 2006).

pH decreased up to 0.6 units in the immediate vicinity of the roots within an average radius of 3.7 mm from the root surface, demonstrating that *S. anglica* exerts control of pH in its rhizosphere. pH in the rhizosphere did not change with altering light conditions and corresponding variation in sediment oxygenation. These findings are in line with Ca çador et al. (1996) reporting pH to be lower in *Spartina maritima* rhizospheres than in closely located unvegetated sediments, and Catallo (1999) finding pH in *Spartina alterniflora* rhizospheres to be stable during 2 days of continuous measurements and independent of strong daily redox fluctuations from -150 to -60 mV. Multiple factors induced by roots lower pH of the surrounding sediment, but processes without diurnal changes occur to be more relevant in determining rhizosphere pH. Oxidative acidification as well as ammonium uptake are highly affected by O_2_ availability (Morris and Dacey, 1984; Bradley and Morris, 1991). Consequently, these processes are expected to have limited influence on pH in these studies, as pH showed no diurnal variation and thereby was unaffected by diurnal changes in sediment oxygenation. Root exudation of low molecular weight organic acids occur in *Spartina* dominated marshes, but the turnover of these compounds is very low (Hines et al., 1994). Consequently, pH changes facilitated by root exudation is not expected to have marked diurnal fluctuations. Furthermore, CO_2_ accumulation around the roots, which was directly measured in this study, showed no response to changing light conditions. Hence, the latter two processes are likely to be the primary cause of the pH drop observed in the vicinity of the roots.

Carbon dioxide accumulated around the roots within an average radius of 12.6 mm from the root surface, which demonstrates that respiration of roots and root-associated microbes result in a marked spatial heterogeneity of CO_2_ in the rhizosphere. Previous studies have also shown large spatial variation in sediment CO_2_ concentrations of *S. anglica* inhabited marshes. In a microcosm experiment, Gribsholt and Kristensen (2002) observed accumulation of pore-water CO_2_ over time, but with considerable spatial and seasonal variation in the CO_2_ concentration, characterized by a subsurface maximum of 20 mM at 20 cm depth during the summer months. Our study also showed a large heterogeneity in the spatial CO_2_ distribution and, in turn, clearly demonstrated how the accumulation of CO_2_ in the sediment is linked to the spatial location of the roots, where the CO_2_ concentration was more than three times the concentration of the bulk sediment.

The release of O_2_ and the exudation of low molecular weight organic compounds as microbial substrate can facilitate stimulation of the microbial community, resulting in increased respiratory activity in the vicinity of roots (Megonigal et al., 2004; Mueller et al., 2016). However, the CO_2_ accumulation did not vary with changing light conditions and the concomitant changes in O_2_ release. Thus, CO_2_ accumulation is expected to be primarily fueled by respiration of the belowground biomass. Root respiration is likely to be independent of photosynthetic activity. Previous studies in *Spartina* grasses have shown that root respiration was unaffected by illumination of the aboveground biomass with similar respiration rates during day and night (Morris and Dacey, 1984). Furthermore, our investigations were conducted at constant temperatures in light and darkness, and consequently temperature, which is a primary driver of diurnal CO_2_ fluxes in vegetated sediments (Parkin and Kaspar, 2003), does not play a role in our investigations.

### Cumulative Rhizosphere Effects on Sediment Biogeochemistry

In this study, we focus exclusively on single roots and their impact on abiotic parameters in the surrounding sediment. However, inside a rhizosphere with multiple closely located roots simultaneously exerting their impact on the sediment, the cumulative effect may be more pronounced than the effects of single roots shown in this study. While sediment oxygenation only affects a small area around the root and has a limited effect on the bulk anoxic sediment in *S. anglica* rhizospheres (Koop-Jakobsen et al., 2017), pH is affected in a larger area around the root, and cumulative effects are more likely. Patrick and Delaune (1977) showed a pH profile of a *S. alterniflora* marsh with distinct lowering of pH where the belowground biomass was dense. However, this effect is not always shown (Koretsky et al., 2008; Bu et al., 2015) and may depend on root density and pore-water buffering capacity of the individual marsh systems.

Considering the relatively large area of influence of CO_2_ (radius 12.6 mm), a cumulative effect is expected to enhance the CO_2_ level of the entire rhizosphere. Previous studies have shown CO_2_ accumulation in marsh rhizospheres resulting in a strong efflux to the atmosphere (Howes et al., 1985).

Our study demonstrates that there are marked differences among individual plants in the dynamics of oxic root zones during alternating light–dark conditions. These differences may also influence the cumulative effect resulting in fewer oxic root zones at night than during the day, which may have a marked impact on the cumulative diurnal dynamics of biogeochemical cycles in the rhizosphere. It also shows the importance of using individual plants for further studies on rhizosphere biogeochemistry in wetland plants in order to capture potential intraspecific differences. However, in the salt-marsh pioneer zones of the European Wadden Sea, *S. anglica* primarily grows as monocultures with a high degree of connectivity between shoots. Therefore, intraspecific variation may be less pronounced in the field, and more studies are needed on the connectivity in natural *S. anglica* settings and its importance for belowground rhizosphere processes.

### Planar Optodes – Methodological Considerations

Planar optode technology has its strength in the clear 2D visualization of the heterogeneous distribution of O_2_, protons (pH), and CO_2_. However, because this optode technique relies on distribution of the analyte up against a planar surface, the distribution of the analyte gets skewed. Since the side of the rhizobox acts as an impermeable barrier, the analytes generated along the root [in this case O_2_, protons (pH), and CO_2_] will accumulate more due to the hindered movement in the direction of the optode foil. This causes an enlargement of the plane of the analyte plume (Polerecky et al., 2006) relative to a root system with unrestricted movement in all directions around the root. Despite this systematic drawback, the planar optode technique still provides a unique opportunity to get insight into the spatial and temporal dynamics of O_2_, pH, and CO_2_ and to visualize the physicochemical interaction between roots and their surroundings, which no other method can provide. In general, the planar optode technique was found to be an excellent tool for visualizing and monitoring the dynamic changes of O_2_, pH, and CO_2_ around plant roots in sediments. The addition of CO_2_ to the sweep of parameters that can be visualized by planar optode technology provides now an opportunity for renewed insights into the rhizosphere processes driving respiration, decomposition, and carbon turnover in wetland rhizospheres.

### Perspective – Cross-Species Comparisons in *Spartina* spp. and Other Wetland Plants

In the discussion and throughout the paper, we compare our results to other studies carried out in *Spartina* dominated salt marshes, most of which are *S. alterniflora* and *S. maritima* dominated. *S. anglica*, which was investigated in this study, is a hybrid of *S. maritima* and *S. alterniflora* that inhabits the same niches and has the same traits in terms of gas transport. Consequently, generalized cross species comparison in regard to plant-sediment interactions are useful. Furthermore, the high capability to translocate oxygen to the rhizosphere is a trait that also other prominent wetland plants share, such as *Phragmites australis* and *Typha* spp. (Armstrong et al., 2000; Wießner et al., 2002), and it may even be considered as a diagnostic trait for aggressive wetland invaders (Mozdzer et al., 2016; Bernal et al., 2017). Thus, the findings presented here can also help reveal insight into mechanisms driving species distribution and invasion in wetland ecosystems.

## Author Contributions

KK-J conceived this research idea, analyzed the data, and wrote the manuscript. PM designed the experiments in collaboration with KK-J, performed the experiments, and was the primary editor of the manuscript. GL and RM provided the equipment and provided – as part of this research – a further development of the technological procedures for image acquisition and image processing that rendered this research project possible. KJ provided the infrastructure necessary for conduction of the experiments. GL, RM, and KJ provided valuable advice on the manuscript. All authors read and approved the manuscript.

## Conflict of Interest Statement

The sensor company, PreSens GmbH provided the planar optode equipment for this study, and optimization of technological procedures on image acquisition and image processing was developed in a collaborative effort with PreSens GmbH. However, PreSens GmbH had no restrictive rights in regard to this publication, and all final decisions on the content of this manuscript was solely the responsibility of the first author. The other authors declare that the research was conducted in the absence of any commercial or financial relationships that could be construed as a potential conflict of interest.

## References

[B1] Armstrong W. (1971). Radial oxygen losses from intact rice roots as affected by distance from the apex, respiration and waterlogging. *Physiol. Plant.* 25 192–197. 10.1111/j.1399-3054.1971.tb01427.x

[B2] Armstrong W. Cousins D. Armstrong J. Turner D. W. Beckett P. M. (2000). Oxygen distribution in wetland plant roots and permeability barriers to gas-exchange with the rhizosphere: a microelectrode and modelling study with *Phragmites australis*. *Ann. Bot.* 86 687–703. 10.1006/anbo.2000.1236

[B3] Askaer L. Elberling B. Glud R. N. Kühl M. Lauritsen F. R. Joensen H. P. (2010). Soil heterogeneity effects on O_2_ distribution and CH_4_ emissions from wetlands: in situ and mesocosm studies with planar O_2_ optodes and membrane inlet mass spectrometry. *Soil Biol. Biochem.* 42 2254–2265. 10.1016/j.soilbio.2010.08.026

[B4] Begg C. B. M. Kirk G. J. D. Mackenzie A. F. Neue H. U. (1994). Root-induced iron oxidation and pH changes in the lowland rice rhizosphere. *New Phytol.* 128 469–477. 10.1111/j.1469-8137.1994.tb02993.x 33874563

[B5] Bernal B. Megonigal J. P. Mozdzer T. J. (2017). An invasive wetland grass primes deep soil carbon pools. *Glob. Change Biol.* 23 2104–2116. 10.1111/gcb.13539 27779794

[B6] Bloom A. J. Meyerhoff P. A. Taylor A. R. Rost T. L. (2002). Root development and absorption of ammonium and nitrate from the rhizosphere. *J. Plant Growth Regul.* 21 416–431. 10.1007/s00344-003-0009-8

[B7] Blossfeld S. (2013). Light for the dark side of plant life: –Planar optodes visualizing rhizosphere processes. *Plant Soil* 369 29–32. 10.1007/s11104-013-1767-0

[B8] Blossfeld S. Gansert D. (2007). A novel non-invasive optical method for quantitative visualization of pH dynamics in the rhizosphere of plants. *Plant Cell Environ.* 30 176–186. 10.1111/j.1365-3040.2006.01616.x 17238909

[B9] Blossfeld S. Gansert D. Thiele B. Kuhn A. J. Loesch R. (2011). The dynamics of oxygen concentration, pH value, and organic acids in the rhizosphere of *Juncus* spp. *Soil Biol. Biochem.* 43 1186–1197. 10.1016/j.soilbio.2011.02.007

[B10] Blossfeld S. Schreiber C. M. Liebsch G. Kuhn A. J. Hinsinger P. (2013). Quantitative imaging of rhizosphere pH and CO_2_ dynamics with planar optodes. *Ann. Bot.* 112 267–276. 10.1093/aob/mct047 23532048 PMC3698388

[B11] Bradley P. M. Morris J. T. (1990). Influence of oxygen and sulfide concentration on nitrogen uptake kinetics in *Spartina alterniflora*. *Ecology* 71 282–287. 10.2307/1940267

[B12] Bradley P. M. Morris J. T. (1991). The influence of salinity on the kinetics of NH4^+^ uptake in *Spartina alterniflora*. *Oecologia* 85 375–380. 10.1007/BF00320613 28312042

[B13] Bu N. Qu J. Li Z. Li G. Zhao H. Zhao B. (2015). Effects of *Spartina alterniflora* invasion on soil respiration in the Yangtze River estuary, China. *PLoS One* 10:e0121571. 10.1371/journal.pone.0121571 25799512 PMC4370568

[B14] Ca çador I. Vale C. Catarino F. (1996). Accumulation of Zn, Pb, Cu, Cr and Ni in sediments between roots of the Tagus estuary salt marshes, Portugal. *Estuar. Coast. Shelf Sci.* 42 393–403. 10.1006/ecss.1996.0026

[B15] Catallo W. J. (1999). *Hourly and Daily Variation of Sediment Redox Potential in Tidal Wetland Sediments.* Biological Division Science Report USGS/BRD/BSR-1999–1991 Reston, VA: U.S. Geological Survey.

[B16] Ehrenfeld J. G. Ravit B. Elgersma K. (2005). Feedback in the plant-soil system. *Annu. Rev. Environ. Resour.* 30 75–115. 10.1146/annurev.energy.30.050504.144212

[B17] Frederiksen M. S. Glud R. N. (2006). Oxygen dynamics in the rhizosphere of *Zostera marina*: a two-dimensional planar optode study. *Limnol. Oceanogr.* 51 1072–1083. 10.4319/lo.2006.51.2.1072

[B18] Gribsholt B. Kristensen E. (2002). Effects of bioturbation and plant roots on salt marsh biogeochemistry: a mesocosm study. *Mar. Ecol. Prog. Ser.* 241 71–87. 10.3354/meps241071

[B19] Hines M. E. Banta G. T. Giblin A. E. Hobbie J. E. (1994). Acetate concentrations and oxidation in salt-marsh sediments. *Limnol. Oceanogr.* 39 140–148. 10.4319/lo.1994.39.1.0140

[B20] Hinsinger P. Bengough A. G. Vetterlein D. Young I. M. (2009). Rhizosphere: biophysics, biogeochemistry and ecological relevance. *Plant Soil* 321 117–152. 10.1007/s11104-008-9885-9

[B21] Hinsinger P. Plassard C. Tang C. Jaillard B. (2003). Origins of root-mediated pH changes in the rhizosphere and their responses to environmental constraints: a review. *Plant Soil* 248 43–59. 10.1023/A:1022371130939

[B22] Howes B. L. Dacey J. W. H. Teal J. M. (1985). Annual carbon mineralization and belowground production of *Spartina alterniflora* in a New England salt marsh. *Ecology* 66 595–605. 10.2307/1940408

[B23] Howes B. L. Teal J. M. (1994). Oxygen loss from *Spartina alterniflora* and its relationship to salt marsh oxygen balance. *Oecologia* 97 431–438. 10.1007/BF00325879 28313730

[B24] Koop-Jakobsen K. Fischer J. Wenzhöfer F. (2017). Survey of sediment oxygenation in rhizospheres of the saltmarsh grass - *Spartina anglica*. *Sci. Total Environ.* 589 191–199. 10.1016/j.scitotenv.2017.02.147 28262356

[B25] Koop-Jakobsen K. Wenzhöfer F. (2015). The dynamics of plant-mediated sediment oxygenation in *Spartina anglica* rhizospheres—a planar optode study. *Estuar. Coasts* 38 951–963. 10.1007/s12237-014-9861-y

[B26] Koretsky C. M. Haveman M. Cuellar A. Beuving L. Shattuck T. Wagner M. (2008). Influence of *Spartina* and *Juncus* on saltmarsh sediments. I. Pore water geochemistry. *Chem. Geol.* 255 87–99. 10.1007/s00248-015-0651-2 26271740

[B27] Lai W.-L. Zhang Y. Chen Z.-H. (2012). Radial oxygen loss, photosynthesis, and nutrient removal of 35 wetland plants. *Ecol. Eng.* 39 24–30. 10.1016/j.watres.2011.05.002 21640369

[B28] Larsen M. Santner J. Oburger E. Wenzel W. W. Glud R. N. (2015). O_2_ dynamics in the rhizosphere of young rice plants (*Oryza sativa* L.) as studied by planar optodes. *Plant Soil* 390 279–292. 10.1007/s11104-015-2382-z 26166902 PMC4495287

[B29] Lee R. W. (1999). Oxidation of sulfide by *Spartina alterniflora* roots. *Limnol. Oceanogr.* 44 1155–1159. 10.4319/lo.1999.44.4.1155 23534207

[B30] Lenzewski N. Mueller P. Meier R. J. Liebsch G. Jensen K. Koop-Jakobsen K. (2018). Dynamics of oxygen and carbon dioxide in rhizospheres of *Lobelia dortmanna* – a planar optode study of belowground gas exchange between plants and sediment. *New Phytol.* 218 131–141. 10.1111/nph.14973 29314005

[B31] Megonigal J. P. Hines M. E. Visscher P. T. (2004). “Anaerobic metabolism: linkages to trace gases and aerobic processes,” in *Biogeochemistry* ed. Schlesinger W. H. (Oxford: Elsevier-Pergamon).

[B32] Mendelssohn I. A. Morris J. T. (2002). “Eco-physiological controls on the productivity of *Spartina alterniflora* Loisel,” in *Concepts and Controversies in Tidal Marsh Ecology* eds Weinstein M. P. Kreeger D. A. (Dordrecht: Kluwer Academic Publishers).

[B33] Millero F. J. Graham T. B. Huang F. Bustos-Serrano H. Pierrot D. (2006). Dissociation constants of carbonic acid in seawater as a function of salinity and temperature. *Mar. Chem.* 100 80–94. 10.1016/j.marchem.2005.12.001

[B34] Minett D. A. Cook P. L. M. Kessler A. J. Cavagnaro T. R. (2013). Root effects on the spatial and temporal dynamics of oxygen in sand-based laboratory-scale constructed biofilters. *Ecol. Eng.* 58 414–422. 10.1016/j.ecoleng.2013.06.028

[B35] Morris J. T. Dacey J. W. H. (1984). Effects of O_2_ on ammonium uptake and root respiration by *Spartina alterniflora*. *Am. J. Bot.* 71 979–985. 10.1002/j.1537-2197.1984.tb14164.x

[B36] Mozdzer T. J. Langley J. A. Mueller P. Megonigal J. P. (2016). Deep rooting and global change facilitate spread of invasive grass. *Biol. Invasions* 18 2619–2631. 10.1007/s10530-016-1156-8

[B37] Mueller P. Jensen K. Megonigal J. P. (2016). Plants mediate soil organic matter decomposition in response to sea level rise. *Glob. Change Biol.* 22 404–414. 10.1111/gcb.13082 26342160

[B38] Neumann G. George T. Plassard C. (2009). Strategies and methods for studying the rhizosphere—the plant science toolbox. *Plant Soil* 321 431–456. 10.1007/s11104-009-9953-9

[B39] Parkin T. B. Kaspar T. C. (2003). Temperature controls on diurnal carbon dioxide flux. *Soil Sci. Soc. Am. J.* 67 1763–1772. 10.2136/sssaj2003.1763

[B40] Patrick W. H. Delaune R. D. (1977). Chemical and biological redox systems affecting nutrient availability in the coastal wetlands. *Geosci. Man* 18 131–137.

[B41] Pezeshki S. R. (2001). Wetland plant responses to soil flooding. *Environ. Exp. Bot.* 46 299–312. 10.1016/S0098-8472(01)00107-1

[B42] Polerecky L. Volkenborn N. Stief P. (2006). High temporal resolution oxygen imaging in bioirrigated sediments. *Environ. Sci. Technol.* 40 5763–5769. 10.1021/es060494l 17007138

[B43] Rozema J. Luppes E. Broekman R. (1985). Differential response of salt-marsh species to variation of iron and manganese. *Vegetatio* 62 293–301. 10.1007/BF00044756

[B44] Santner J. Larsen M. Kreuzeder A. Glud R. N. (2015). Two decades of chemical imaging of solutes in sediments and soils – a review. *Anal. Chim. Acta* 878 9–42. 10.1016/j.aca.2015.02.006 26002324

[B45] Schreiber C. M. Zeng B. Blossfeld S. Rascher U. Kazda M. Schurr U. (2012). Monitoring rhizospheric pH, oxygen, and organic acid dynamics in two short-time flooded plant species. *J. Plant Nutr. Soil Sci.* 175 761–768. 10.1002/jpln.201000427

[B46] Tschiersch H. Liebsch G. Stangelmayer A. Ljudmilla B. Rolletschek H. (2011). “Planar oxygen sensors for non invasive imaging in experimental biology,” in *Microsensors* ed. Minin I. (Rijeka: Intechopen) 281–294.

[B47] Wang X. D. Meier R. J. Link M. Wolfbeis O. S. (2010). Photographing oxygen distribution. *Angew. Chem. Int. Ed. Engl.* 49 4907–4909. 10.1002/anie.201001305 20540131

[B48] Wießner A. Kuschk P. Stottmeister U. (2002). Oxygen release by roots of *Typha latifolia* and *Juncus effusus* in laboratory hydroponic systems. *Eng. Life Sci.* 22 209–216. 10.1002/1521-3846(200205)22:1/2<209::AID-ABIO209>3.0.CO;2-O

[B49] Winkel A. Colmer T. D. Pedersen O. (2011). Leaf gas films of *Spartina anglica* enhance rhizome and root oxygen during tidal submergence. *Plant Cell Environ.* 34 2083–2092. 10.1111/j.1365-3040.2011.02405.x 21819414

[B50] Wolf A. A. Drake B. G. Erickson J. E. Megonigal J. P. (2007). An oxygen-mediated positive feedback between elevated carbon dioxide and soil organic matter decomposition in a simulated anaerobic wetland. *Glob. Change Biol.* 13 2036–2044. 10.1111/j.1365-2486.2007.01407.x

